# Design Space Development for the Extraction Process of Danhong Injection Using a Monte Carlo Simulation Method

**DOI:** 10.1371/journal.pone.0128236

**Published:** 2015-05-28

**Authors:** Xingchu Gong, Yao Li, Huali Chen, Haibin Qu

**Affiliations:** Pharmaceutical Informatics Institute, College of Pharmaceutical Sciences, Zhejiang University, Hangzhou 310058, China; Politehnica University of Bucharest, ROMANIA

## Abstract

A design space approach was applied to optimize the extraction process of Danhong injection. Dry matter yield and the yields of five active ingredients were selected as process critical quality attributes (CQAs). Extraction number, extraction time, and the mass ratio of water and material (W/M ratio) were selected as critical process parameters (CPPs). Quadratic models between CPPs and CQAs were developed with determination coefficients higher than 0.94. Active ingredient yields and dry matter yield increased as the extraction number increased. Monte-Carlo simulation with models established using a stepwise regression method was applied to calculate the probability-based design space. Step length showed little effect on the calculation results. Higher simulation number led to results with lower dispersion. Data generated in a Monte Carlo simulation following a normal distribution led to a design space with a smaller size. An optimized calculation condition was obtained with 10000 simulation times, 0.01 calculation step length, a significance level value of 0.35 for adding or removing terms in a stepwise regression, and a normal distribution for data generation. The design space with a probability higher than 0.95 to attain the CQA criteria was calculated and verified successfully. Normal operating ranges of 8.2-10 g/g of W/M ratio, 1.25-1.63 h of extraction time, and two extractions were recommended. The optimized calculation conditions can conveniently be used in design space development for other pharmaceutical processes.

## Introduction

Danhong injection is a botanical injection used in the treatment of coronary heart disease, angina, myocardial infarction, and cerebral diseases [1]. The injection is made from Salviae miltiorrhizae Radix et Rhizoma (Danshen) and Carthami Flos (Honghua) using unit operations of extraction, concentration, ethanol precipitation, adsorption, etc. The water extraction process of mixed Danshen and Honghua affects both the drug efficacy and the drug safety of the Danhong injection. The optimization of the extraction process will contribute to an increase in the batch-to-batch consistency of the Danhong injection.

Quality by design concept has become an essential part of the modern approach to ensure pharmaceutical product quality [2, 3]. Design space development plays an important role in the implementation of the quality by design concept [4, 5]. To develop a reliable design space, process critical quality attributes (CQAs) and critical process parameters (CPPs) must be identified, and the mathematical models between CPPs and process CQAs must be built. Then, the design space can be calculated and verified. Model building is an important consideration in design space development. Both statistical models and mechanistic models can be used to calculate process design space [6, 7]. However, for the separation processes in the manufacture of botanical drugs, building mechanistic models is usually very difficult because of the complex composition and lack of fundamental physical and chemical data. Therefore, statistical models between CPPs and process CQAs are usually used. Typically, quadratic models containing linear terms, nonlinear terms, and interaction terms are applied. These quadratic models are simple and can be a good approximation of the true relationships. The models can be developed after the design of the experiments (DOE). The response surface DOE such as central composite design and Box-Behnken design are widely used to establish the quadratic models [6, 8–10]. To remove insignificant terms, stepwise regression is usually used in model building. The significance level values in stepwise regression should be selected carefully.

Recently, probability-based design space has come to the forefront because it can provide the assurance of design space to meet all process specifications. Rozet et al. reinterpreted the definition of design space, which was “a multivariate domain of input factors ensuring that critically chosen responses are included within predefined limits with an acceptable level of probability” [11]. Bayesian modeling, bootstrapping techniques, and Monte Carlo simulation are three methods to calculate the probability of meeting the specifications imposed on the CQAs [11]. Peterson et al. gave several examples using the Bayesian predictive method to calculate the probability-based design space [4, 12]. Our group developed design spaces for the ethanol precipitation process [13], the water precipitation process [14], and the extraction process [15] using a Monte Carlo simulation method. The Monte Carlo simulation method was also used successfully in the design space development for several analytical methods [16–19]. In the Monte Carlo simulation, random data following a given distribution will be generated. The data distribution type and simulation number are important factors that may affect the calculation results. However, these calculation parameters have not been published.

In this work, process CQAs and CPPs of the extraction process were selected. Quadratic models were built. Simulation number, calculation step length, data distribution type, and the significance level value of the stepwise regression were optimized in the Monte Carlo simulation. Different simulation results were calculated and compared. The probability-based design space was obtained using the optimized Monte Carlo simulation conditions. Then, the design space was verified. The characteristics of this calculation method are also discussed.

## Experimental Section

### 2.1 Materials and Chemicals

Danshen was purchased from Nepstar Drugstore (Hangzhou, Zhejiang, China). Honghua was purchased from Daily Healthy Drugstore (Hangzhou, Zhejiang, China). No specific permission was required for the field studies described in this paper. The locations are neither privately owned nor protected by the Chinese government. No endangered or protected species were sampled.

Standard substances, including rosmarinic acid, Danshensu, and lithospermic acid, were purchased from Winherb Medical S&T Development Co., Ltd. (Shanghai, China). Salvianolic acid B was purchased from Chengdu Biopurify Phytochemicals Ltd. (Chengdu, Sichuan, China). Hydroxysafflor yellow A was obtained from Aladdin Industrial Inc. (Shanghai, China). Deionized water was produced using an academic water purification system (Milli-Q, Milford, MA, USA). HPLC-grade formic acid was obtained from ROE SCIENTIFIC INC. (Newark, DE, USA). HPLC-grade acetonitrile was purchased from Merck (Darmstadt, Germany). HPLC-grade ammonium formate was obtained from Alfa Aesar China (Tianjin, China) Co., Ltd. All materials were used as received without any further purification.

### 2.2 Procedure

After 45 g of Danshen and 15 g of Honghua were placed in a round bottom flask, water was added. The flask then was heated using a heating jacket (TC-15, Haining Huaxing Instrument Co. Ltd, China). After reflux extraction for a period of time, the extract was collected by filtration. If the extraction number was more than 1, water was then added to the flask after filtration to extract mixed Danshen and Honghua again. The extracts were mixed before the measurement of active ingredient content and dry matter content.

### 2.3 Design of experiments

In this work, the three parameters of extraction time, extraction number, and the mass ratio of water and material (W/M ratio) were investigated. Table 1 shows the coded and uncoded values of the parameters. The run order is listed in Table 2. The ranges of the three parameters were set based on production experience. After the development of the design space, verification experiments were repeated three times with a reflux time of 1.6 h, W/M ratio of 8.3 g/g, and extraction number of 2.

**Table 1:** Coded and uncoded values of parameters for experimental design.

Parameters	Coded values			
	-1	-0.333	0	1
Extraction time, X_1_ (h)	0.5	1	—	2
W/M ratio, X_2_ (g/g)	6	—	8	10
Extraction number, X_3_	1	—	2	3

**Table 2:** Experimental conditions and results.

Run order	Parameters			Danshensu yield, Y_1_ (mg/g *Danshen*)	Hydroxysafflor yellow A yield, Y_2_ (mg/g *Honghua*)	Rosmarinic acid yield, Y_3_ (mg/g *Danshen*)	Lithospermic acid yield, Y_4_ (mg/g *Danshen*)	Salvianolic acid B yield, Y_5_ (mg/g *Danshen*)	Dry matter yield, Y_6_ (mg/g *Material*)
	X_1_	X_2_	X_3_						
1	1	6	3	2.114	5.822	1.610	2.229	39.76	492.3
2	0.5	10	2	1.209	5.240	2.017	2.025	40.43	466.7
3	0.5	8	3	1.387	5.135	2.206	2.267	45.67	485.8
4	1	10	3	2.340	7.707	2.172	2.630	43.60	537.3
5	1	8	2	1.934	5.786	1.980	2.247	38.38	479.9
6	1	10	1	1.089	5.016	1.565	1.601	29.44	355.8
7	2	8	1	1.674	3.605	1.418	1.628	25.56	340.1
8	1	6	1	0.601	2.588	0.979	0.932	18.76	233.3
9	0.5	8	1	0.596	2.990	1.431	1.235	28.13	318.3
10	2	8	3	3.983	5.719	2.060	2.714	39.61	586.0
11	2	10	2	3.380	4.458	2.057	2.488	35.75	491.7
12	1	8	2	1.715	6.755	2.035	2.183	37.97	437.6
13	0.5	6	2	0.926	3.071	1.858	1.679	36.21	386.0
14	1	8	2	1.834	6.565	2.077	2.304	39.62	485.2
15	2	6	2	3.151	3.614	1.821	2.091	30.58	458.3

### 2.4 Analytical methods

The components Danshensu, hydroxysafflor yellow A, rosmarinic acid, lithospermic acid, and salvianolic acid B were analyzed by HPLC according to the method reported previously [20]. The method is briefly described as follows. The HPLC system HP 1100 series (Agilent Technologies, Waldbronn, Germany) was equipped with the ChemStation software (Agilent Technologies). The separations were carried out on a ZORBAX Eclipse Plus C18 column (100 mm × 4.6 mm, 1.8 μm) with a formic acid-500 mmol·L^-1^ ammonium formate-water solution (0.5:10:90, v/v/v) as mobile phase A and acetonitrile-formic acid solution (100:0.5, v/v) as mobile phase B in a gradient mode at 30°C. The flow rate was 0.5 ml·min^-1^. The gradient program was as follows: 0–10 min, 2–9% B; 10–13 min, 9–10% B; 13–20 min, 10–17% B; 20–37 min, 17–20% B; 37–47 min, 20–25% B; 47–50 min, 25–80% B. The analytical wavelength was set at 280 nm from 0 min to 15.9 min and at 380 nm from 15.9 min to 17.9 min. The wavelength then was set at 280 nm from 17.9 min to 50 min. Dry matter content of the extracts was determined gravimetrically after hot air drying at 105°C for 3 hours.

### 2.5 Data processing

Eqs 1 and 2 were used to calculate the dry matter yield (DMY) and the active ingredient yield (ACY), respectively.

$$DMY=\frac{DM_{ext}\times M_{ext}}{M_{mat}}$$ (1)

where *DM* is dry matter content, and *M* is the mass. Subscripts *ext* and *mat* refer to extract and material, respectively.

$$ACY_{i}=\frac{AC_{ext,i}\times M_{ext}}{M_{mat}}$$ (2)

where *AC* is the active ingredient content and subscript i (i = 1, 2, …, 5) represents Danshensu, hydroxysafflor yellow A, rosmarinic acid, lithospermic acid, and salvianolic acid B, respectively.

The experimental data were analyzed using Design-Expert 8.0.6 software (State-Ease Inc., MN, USA) to obtain response surface models. The mathematical model is shown in Eq 3.

$$Y_{}={\text{a}}_{0}+{\text{a}}_{1}X_{1}+{\text{a}}_{2}X_{2}+{\text{a}}_{3}X_{3}+{\text{a}}_{4}X_{1}X_{2}+{\text{a}}_{5}X_{1}X_{3}+{\text{a}}_{6}X_{2}X_{3}+{\text{a}}_{7}X_{1}^{2}+{\text{a}}_{8}X_{2}^{2}+{\text{a}}_{9}X_{3}^{2}$$ (3)

where a_0_ is a constant, a_1_, a_2_, …, a_9_ are regression coefficients, and Y is a CQA. All the variables were coded before modeling. Insignificant terms were removed using a stepwise regression. The significance levels to remove or add a term were both set to 0.35.

The design space was calculated using a Monte Carlo method with a self-written program of Matlab (R2011b, MathWorks, USA). In the calculations, the uncertainty of the measured data is considered. Random data for the active ingredient content and dry matter content in the supernatant are generated following a given distribution. The given distribution was assumed to be a normal distribution, a lognormal distribution, a square-root-normal distribution, or a reciprocal normal distribution. For the center point, the average value and the corresponding standard deviation value of each process CQA were considered the mean value and standard deviation value of the given distribution. For other experimental points, measured values were considered the mean values in the given distribution. Relative standard deviations (RSDs) of measured data were assumed to be the same as the RSD of the center point. For other experimental points, standard deviation values of the given distribution can then be calculated using the product of the RSD values and the measured values. After the generation of random data for all of the experimental conditions, each data set was applied to develop a model using a stepwise regression. All the models developed were used to predict process CQA values under a given set of conditions. The significance level values were set the same for the move-in and the move-out of model terms. The acceptable level of probability for the design space was set as 0.95. In the investigation of the Monte Carlo simulation conditions, the design space when the extraction number is 2 was calculated as a sample. Two criteria were calculated using coded values of CPPs, namely, average dimensionless size of the design space (ADSS) and the relative standard deviation of the dimensionless design space size (RSDDSS). Calculations were repeated 10 times to obtain the RSDDSS value.

## Results and Discussion

### 3.1 CQA selection

According to the risk assessment results in our previous work [13], the extraction process significantly affects the active ingredient content, fingerprint similarity, and dry matter content of the Danhong injection. Phenolic acids from Danshen such as salvianolic acid B, lithospermic acid, Danshensu, and rosmarinic acid, and flavones from Honghua such as hydroxysafflor yellow A are considered active ingredients of the Danhong injection [21, 22]. These active ingredients can be extracted easily with hot water. However, some active ingredients such as salvianolic acid B or hydroxysafflor yellow A also easily degrade in the extraction process due to hydrolysis or other reactions [23–25]. Accordingly, the yields of active ingredients in the extraction process are prone to fluctuations. Therefore, in this work, the yields of Danshensu, hydroxysafflor yellow A, rosmarinic acid, lithospermic acid, and salvianolic acid B are considered process CQAs. Impurities of saccharides, tannins, pigments, and inorganic salts are extracted simultaneously with the active ingredients, which leads to an increase in dry matter yield. The similarity of fingerprints is affected by both the active ingredient content and the impurity content in injections. Therefore, dry matter yield is considered another process CQA in this work. According to industry experience and literature results [26, 27], the criteria for all of the CQAs were obtained and are listed in Table 3.

**Table 3:** The control limits for process CQAs.

CQAs	Lower limit	Upper limit
Danshensu yield (mg/g *Danshen*)	2.0	3.8
Hydroxysafflor yellow A yield (mg/g *Honghua*)	5.1	7.6
Rosmarinic acid yield (mg/g *Danshen*)	1.8	2.2
Lithospermic acid yield (mg/g *Danshen*)	2.1	2.6
Salvianolic acid B yield (mg/g *Danshen*)	36	45
Dry matter yield (mg/g *material*)	400	550

### 3.2 CPP selection

**Fig 1:**
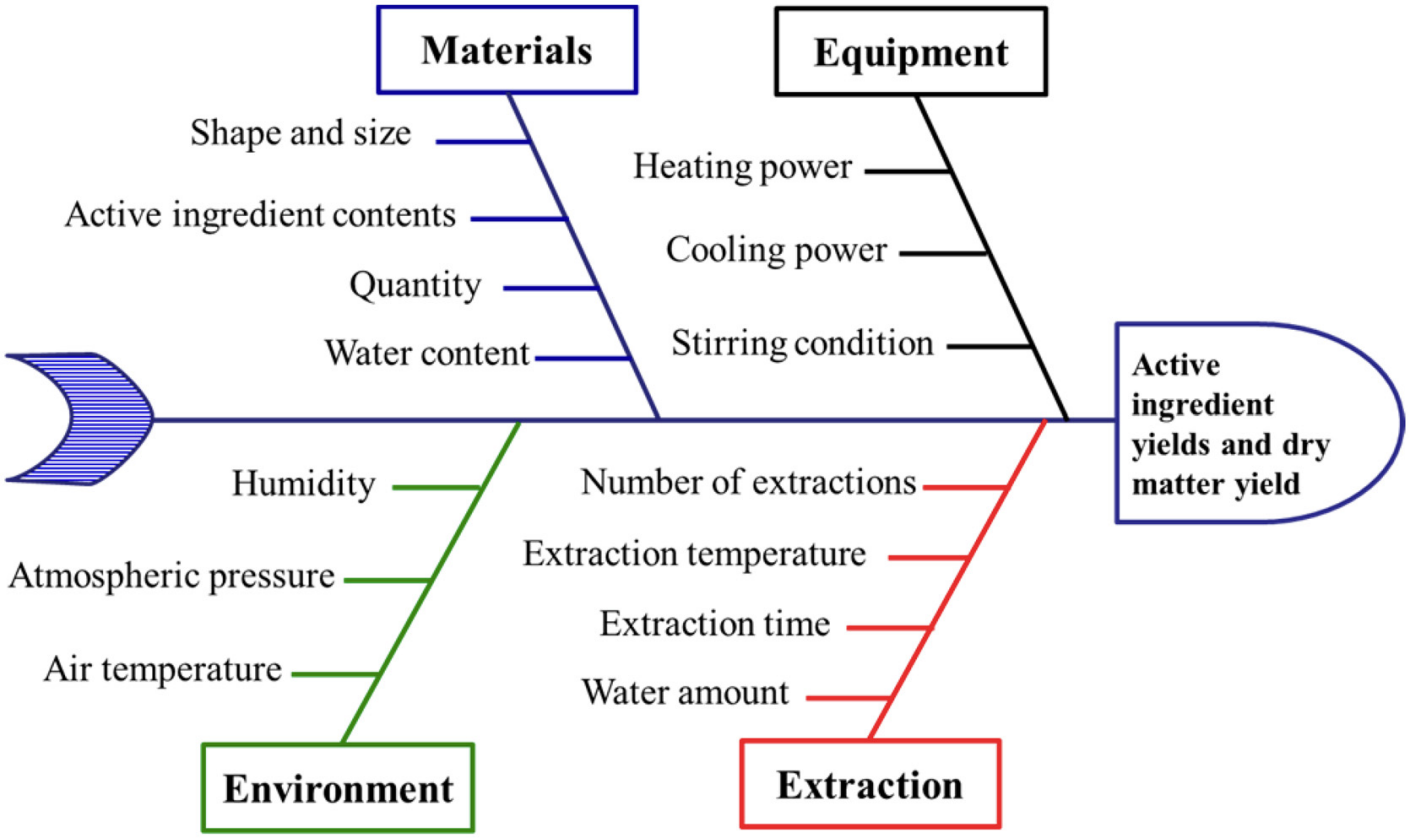
Ishikawa diagram analysis for the extraction process.

Ishikawa diagram analysis was performed to obtain an initial list of potential factors that affect the results of the extraction process, as shown in Fig 1. Four main causes are involved, including environment, material attributes, equipment, and extraction procedure.

Water is the recommended extractant because a higher ethanol content in the mixed ethanol-water solvent results in a lower safflower yellow yield [28]. Extraction time, extraction number, extraction temperature, and solvent amount had a significant impact on safflower yellow yield [28, 29]. Solvent amount, extraction time and extraction number were also considered to be important factors for the extraction of salvianolic acid B from Danshen [30]. Extraction temperature is determined mainly by the solvent composition for reflux extraction at atmospheric pressure. Therefore, the W/M ratio, extraction number, and extraction time are selected as CPPs of the extraction process of Danhong injection.

### 3.3 Effects of CPPs on CQAs

The experimental results of six CQAs in the extraction process are listed in Table 2. Salvianolic acid B yield was between 18.76 and 45.67 mg/g *Danshen*, which was much more than that of Danshensu, rosmarinic acid, or lithospermic acid. Rosmarinic acid yield and lithospermic acid yield were lower than 3 mg/g *Danshen*. Hydroxysafflor yellow A yield varied from 2.588 to 7.707 mg/g *Honghua*. Dry matter yield values varied from 233.3 to 586.0 mg/g *material*. Because dry matter yield values are much higher than the sum of the five active ingredient yields, most of the dry matter extracted appeared to be impurities.

In this work, second-order polynomial models were applied to describe the nonlinear effects of parameters. Models were simplified using stepwise regression. The estimated values of the regression coefficients are listed in Table 4. The determination coefficients (R^2^) are higher than 0.94 for all the models, which means that most variations can be explained by these models. Analysis of Variance (ANOVA) was applied to determine the impact of the W/M ratio, extraction number, and extraction time on all the CQAs. As shown in Table 4, the linear terms of the W/M ratio and extraction number are significant for all the CQAs. The linear term of extraction time is insignificant for the yields of rosmarinic acid and hydroxysafflor yellow A. For the yields of Danshensu, rosmarinic acid, lithospermic acid, and salvianolic acid B, the quadratic term of the extraction number is very significant because the p values are less than 0.01.

**Table 4:** Estimated parameter values, ANOVA results for variables and determined coefficients.

CQAs	Recovery of active ingredients										Dry matter yield	
	Danshensu		Hydroxysafflor yellow A		Rosmarinic acid		Lithospermic acid		Salvianolic acid B			
Terms	Estimate	*p* value	Estimate	*p* value	Estimate	*p* value	Estimate	*p* value	Estimate	*p* value	Estimate	*p* value
Constant	2.166		6.569		2.024		2.329		38.030		474.39	
X_1_	1.012	< 0.0001*					0.214	< 0.0001*	-2.330	0.0002*	26.88	0.0052*
X_2_	0.153	0.0048*	0.855	0.0005*	0.193	0.0004*	0.227	< 0.0001*	2.989	< 0.0001*	32.98	0.0025*
X_3_	0.795	< 0.0001*	1.273	< 0.0001*	0.332	< 0.0001*	0.555	< 0.0001*	8.181	< 0.0001*	109.65	< 0.0001*
X_1_X_2_			-0.365	0.1115							-13.31	0.1836
X_1_X_3_	0.373	0.0001*							-0.968	0.0736	17.50	0.0956
X_2_X_3_	-0.065	0.2792					-0.067	0.0698	-1.709	0.0086*	-19.38	0.0773
X_1_^2^			-1.797	< 0.0001*	0.059	0.3445	-0.115	0.0187				
X_2_^2^			-0.676	0.0148	-0.145	0.0225	-0.143	0.0032*	-2.399	0.0018*	-22.12	0.0577
X_3_^2^	-0.274	0.0015*	-0.409	0.0977	-0.304	0.0003*	-0.253	0.0001*	-3.402	0.0002*	-40.24	0.0053*
R^2^	0.993		0.957		0.946		0.992		0.992		0.983	
R^2^_adj_	0.987		0.924		0.915		0.985		0.983		0.961	

* p < 0.01.

**Fig 2:**
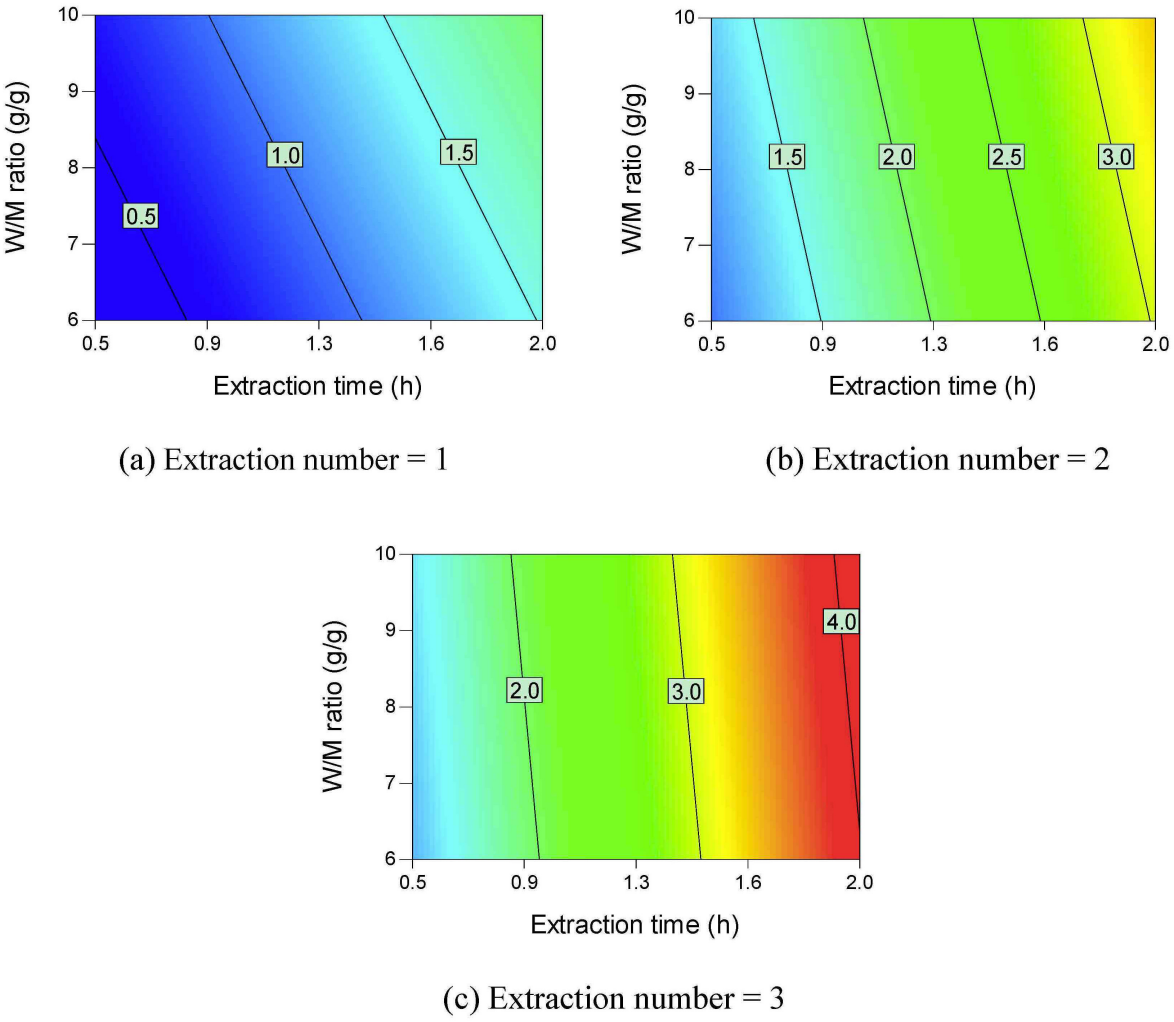
Contour plots of parameter interactions on Danshensu yield.

**Fig 3:**
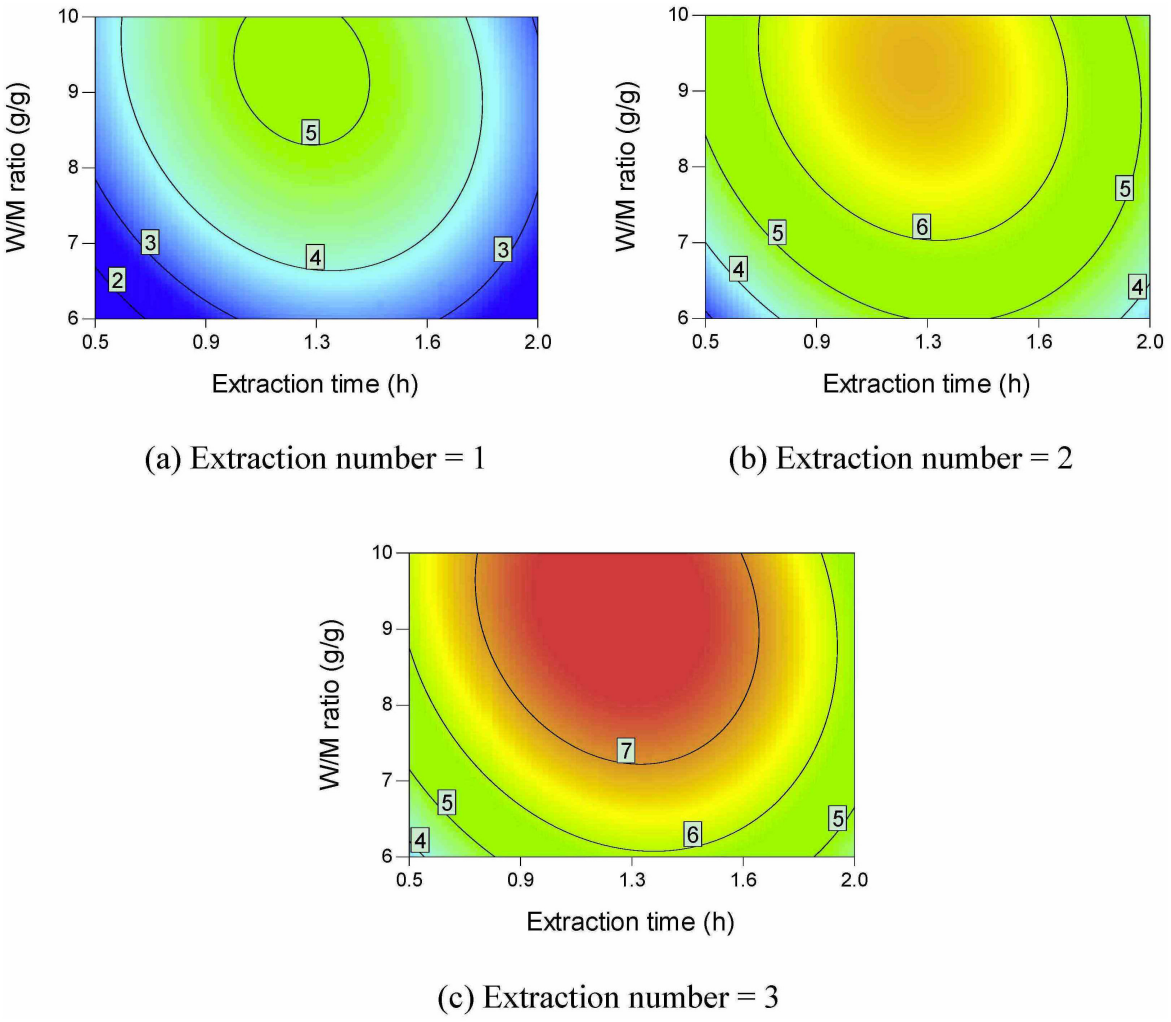
Contour plots of parameter interactions on hydroxysafflor yellow A yield.

**Fig 4:**
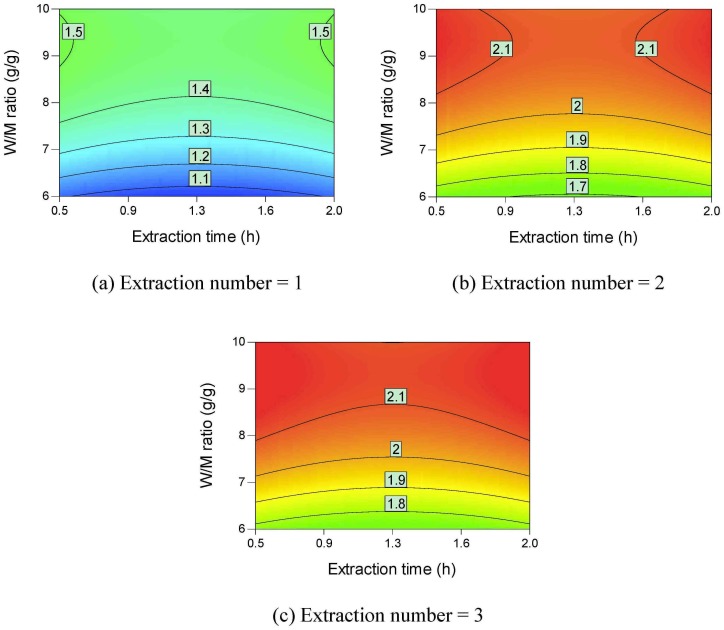
Contour plots of parameter interactions on rosmarinic acid yield.

**Fig 5:**
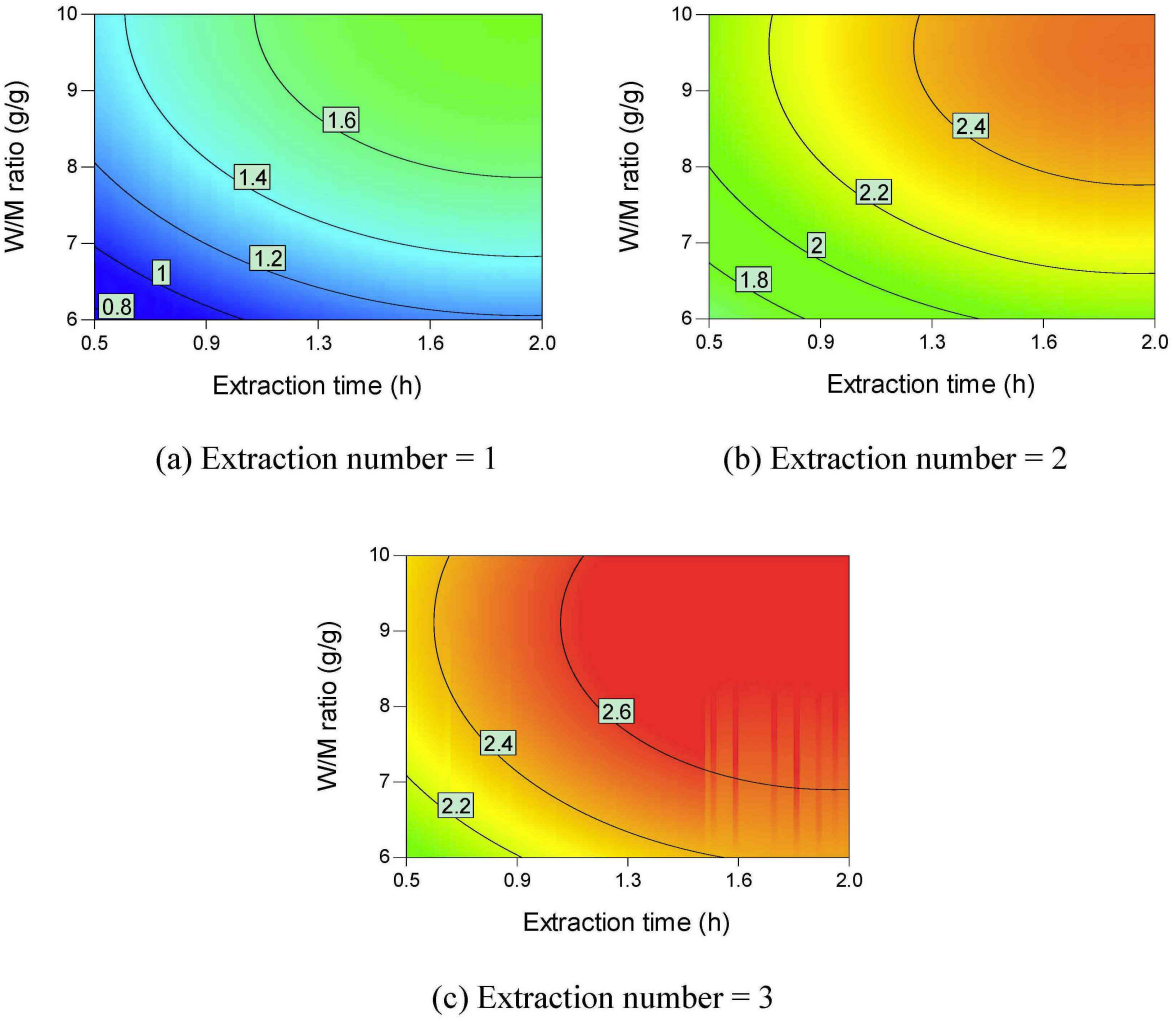
Contour plots of parameter interactions on lithospermic acid yield.

**Fig 6:**
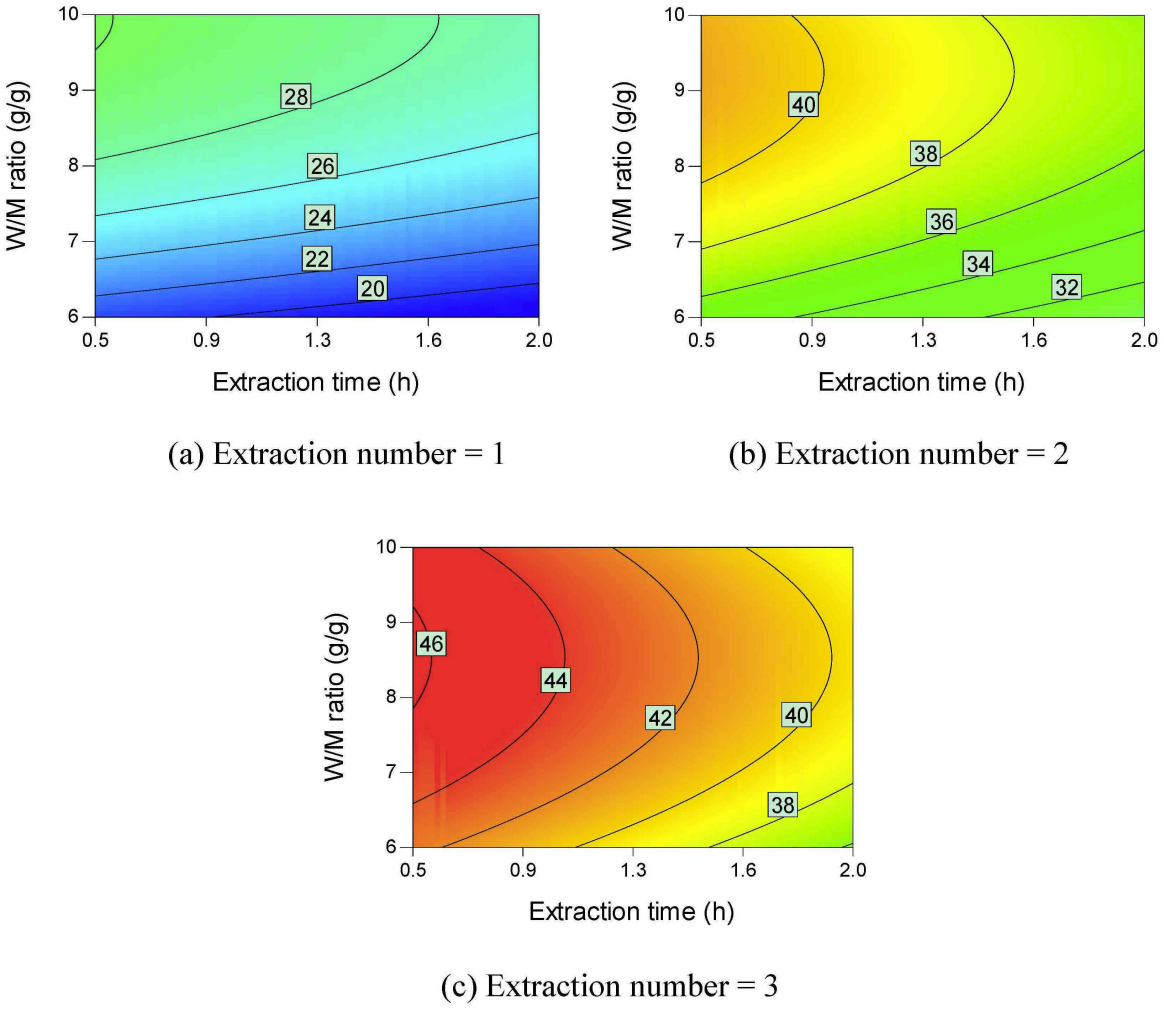
Contour plots of parameter interactions on Salvianolic acid B yield.

Contour plots of the active ingredient yields are shown in Figs 2–6. All the active ingredient yields increase as the extraction number increases. Danshensu yield, lithospermic acid yield, rosmarinic acid yield, and lithospermic acid yield also increase as the W/M ratio increases. Danshensu and lithospermic acid are two of the hydrolyzates of salvianolic acid B [24, 25]. Accordingly, their yields both increase as extraction time increases, as shown in Fig 2 and Fig 5. Salvianolic acid B will hydrolyze and form many compounds [24, 25]. Therefore, the increase in extraction time results in a lower salvianolic acid yield, as shown in Fig 6. Compared with the degradation rate of salvianolic acid B, the degradation rate of rosmarinic acid is much slower [31]. Hydroxysafflor yellow A is a hydrolyzate of anhydrosafflor yellow B [23]. Hydroxysafflor yellow A can also hydrolyze and form *p*-coumaric acid [23]. As shown in Fig 3, when extraction time increases, hydroxysafflor yellow A first increases, then decreases.

**Fig 7:**
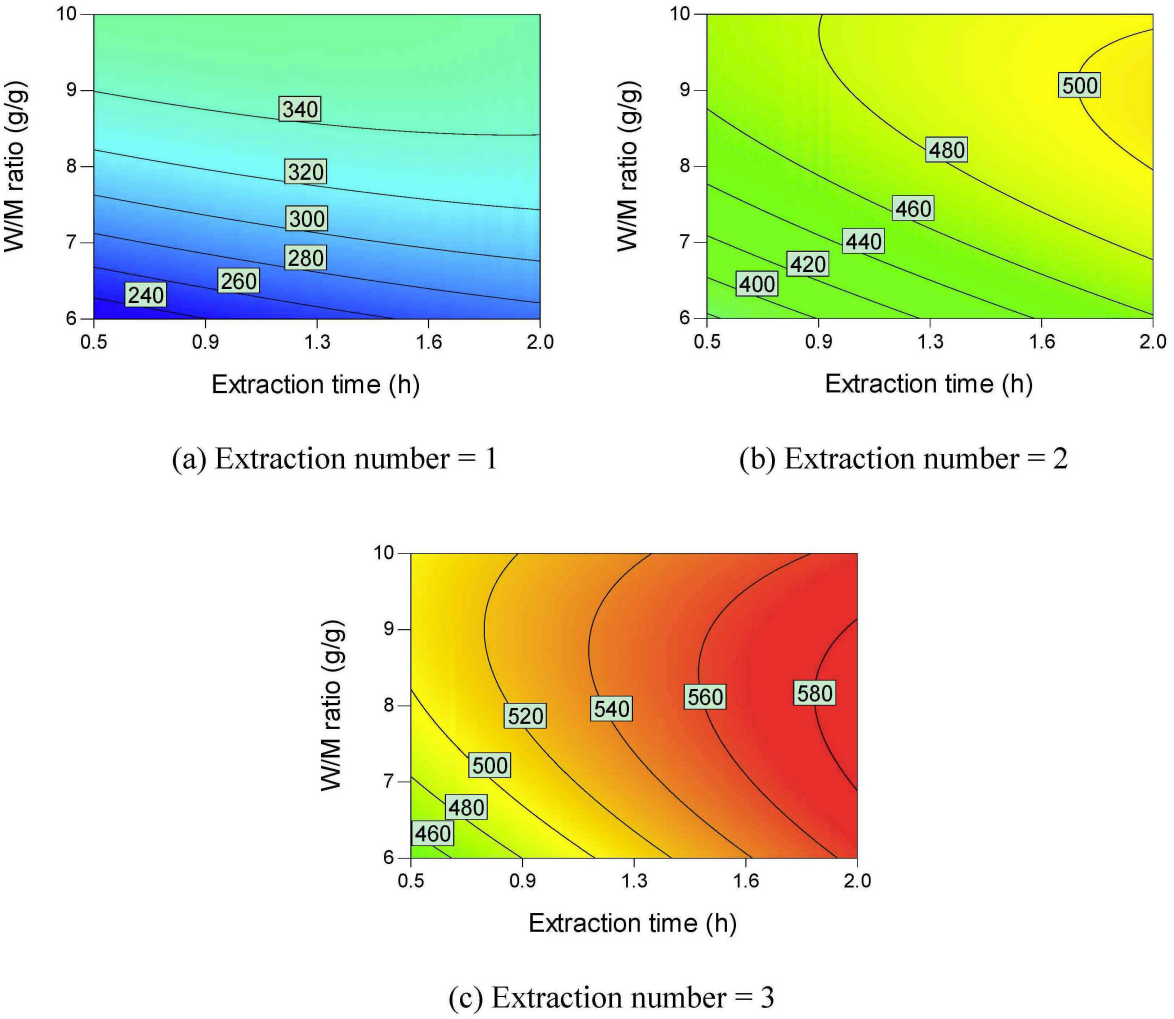
Contour plots of parameter interactions on dry matter yield.

Fig 7 provides the contour plots of parameter interactions on dry matter yield. Dry matter yield increases as the extraction number, W/M ratio, and extraction time increase. Different types of saccharides such as sucrose, fructose, and glucose are found in Danshen [32]. These saccharides are easily soluble in water [33–35]. Phenolic acids usually exist in medicinal plants in their salt forms [36]. The phenolic acids can also be extracted using hot water. Accordingly, the dry matter yield can be higher than 500 mg/g *material*.

### 3.4 Design space development

#### 3.4.1 Simulation number and calculation step length

The results for different simulation numbers and calculation step lengths were calculated. The significance level value was 0.15 for both adding and removing a term. Random data were generated following a normal distribution. The results are shown in Table 5. The variations in calculation step length did not affect ADSS values and RSDDSS values. ADSS changes little as simulation number increases. An increase in simulation number led to a smaller RSDDSS, which means that more reliable simulation results can be obtained. When the simulation number was more than 10000, RSDDSS values were less than 0.5%. Therefore, the simulation number was set at 10000, and the calculation step length was set as 0.01 in the following calculations.

**Table 5:** Effects of simulation number and step length on ADSS and RSDDSS values.

Simulation number	Calculation step length									
	0.008		0.01		0.02		0.03		0.04	
	ADSS	RSDDSS (%)	ADSS	RSDDSS (%)	ADSS	RSDDSS (%)	ADSS	RSDDSS (%)	ADSS	RSDDSS (%)
500	0.923	1.62	0.926	1.63	0.929	1.56	0.918	1.58	0.937	1.39
1000	0.928	1.12	0.928	1.13	0.933	1.10	0.923	1.08	0.942	0.980
5000	0.925	0.530	0.926	0.520	0.930	0.590	0.921	0.570	0.939	0.500
10000	0.924	0.440	0.925	0.460	0.928	0.560	0.920	0.450	0.936	0.430
20000	0.924	0.330	0.925	0.390	0.928	0.490	0.920	0.350	0.937	0.340
30000	0.924	0.230	0.924	0.290	0.927	0.410	0.921	0.190	0.937	0.260

#### 3.4.2 The significance level value in stepwise regression

**Fig 8:**
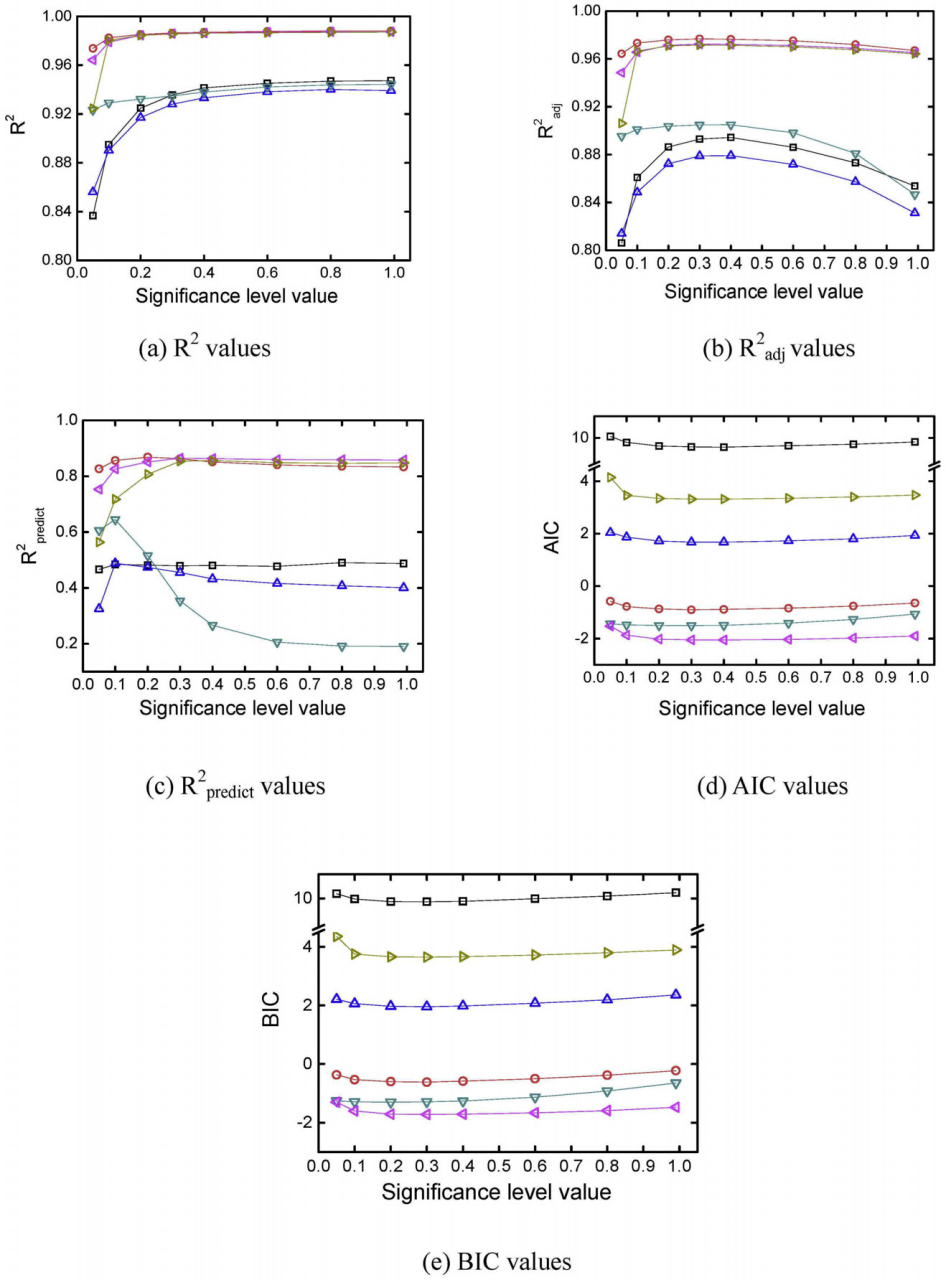
R^2^, R^2^_adj_, R^2^_predict_, AIC, and BIC values after stepwise regression. (☐, dry matter yield; ◯, Danshensu yield; △, hydroxysafflor yellow A yield; ▽, rosmarinic acid yield; ⊲, lithospermic acid yield; ⊳, Salvianolic acid B yield).

Different model criteria of R^2^, the Akaike information criterion, the Bayesian information criterion, R^2^_predict_, and R^2^_adj_ were used to evaluate the quadratic models after stepwise regression with different significance level values. The simulation was repeated 10000 times. The average results for R^2^, the Akaike information criterion, the Bayesian information criterion, R^2^_predict_, and R^2^_adj_ were obtained and are plotted in Fig 8. In Fig 8a, average R^2^ values increase as the significance level value increases for all the models. In Fig 8b and 8c, the average model R^2^_adj_ and R^2^_predict_ values increase first but then decrease as the significance level value increases. Overfitting occurs when too many terms are included in the models. Average Akaike information criterion and Bayesian information criterion values both decrease first and then increase slightly as the significance level value increases, as shown in Fig 8d and 8e. Higher R^2^_adj_, higher R^2^_predict_, lower Akaike information criterion, or lower Bayesian information criterion values are favored in the selection of models. Because the turning points of average R^2^_adj_, Akaike information criterion, and Bayesian information criterion values were all between 0.3 and 0.4, the significance level in the stepwise regression for adding or removing terms was set as 0.35 in following calculations.

#### 3.4.3 Data distribution type

**Fig 9:**
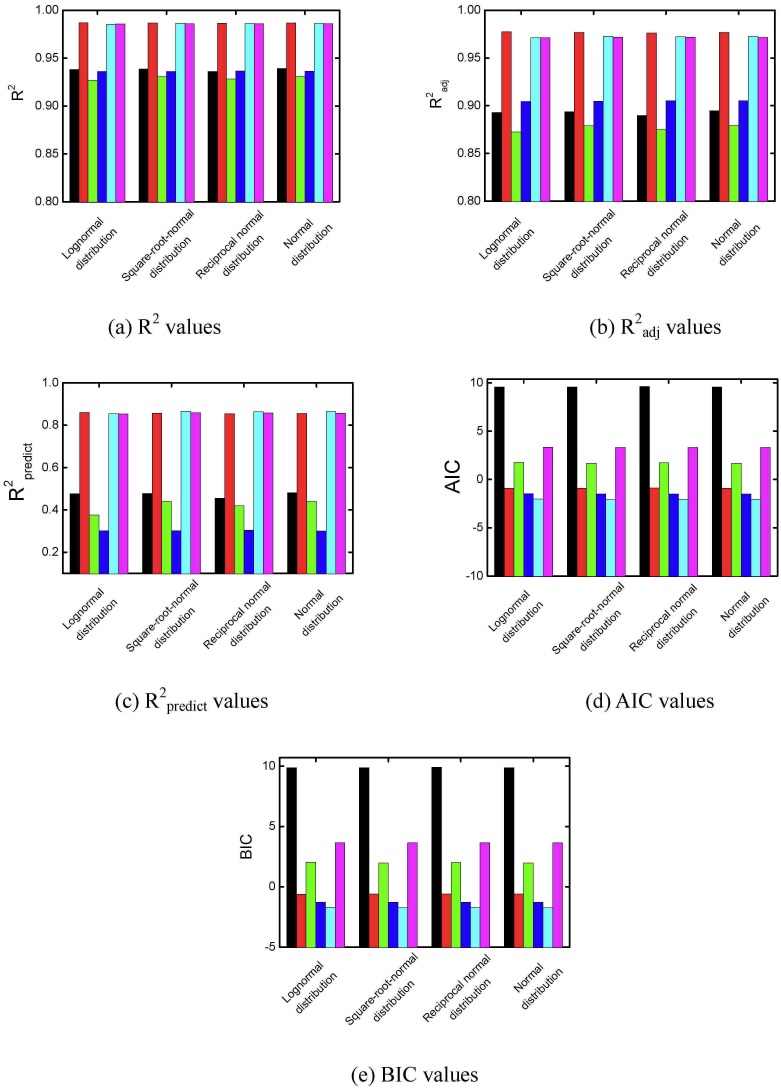
Average values of R^2^, R^2^_adj_, R^2^_predict_, AIC, and BIC after stepwise regression. (Black bar, dry matter yield; Red bar, Danshensu yield, Green bar, hydroxysafflor yellow A yield; Blue bar, rosmarinic acid yield; Cyan bar, lithospermic acid yield; Magenta bar, Salvianolic acid B yield).

**Fig 10:**
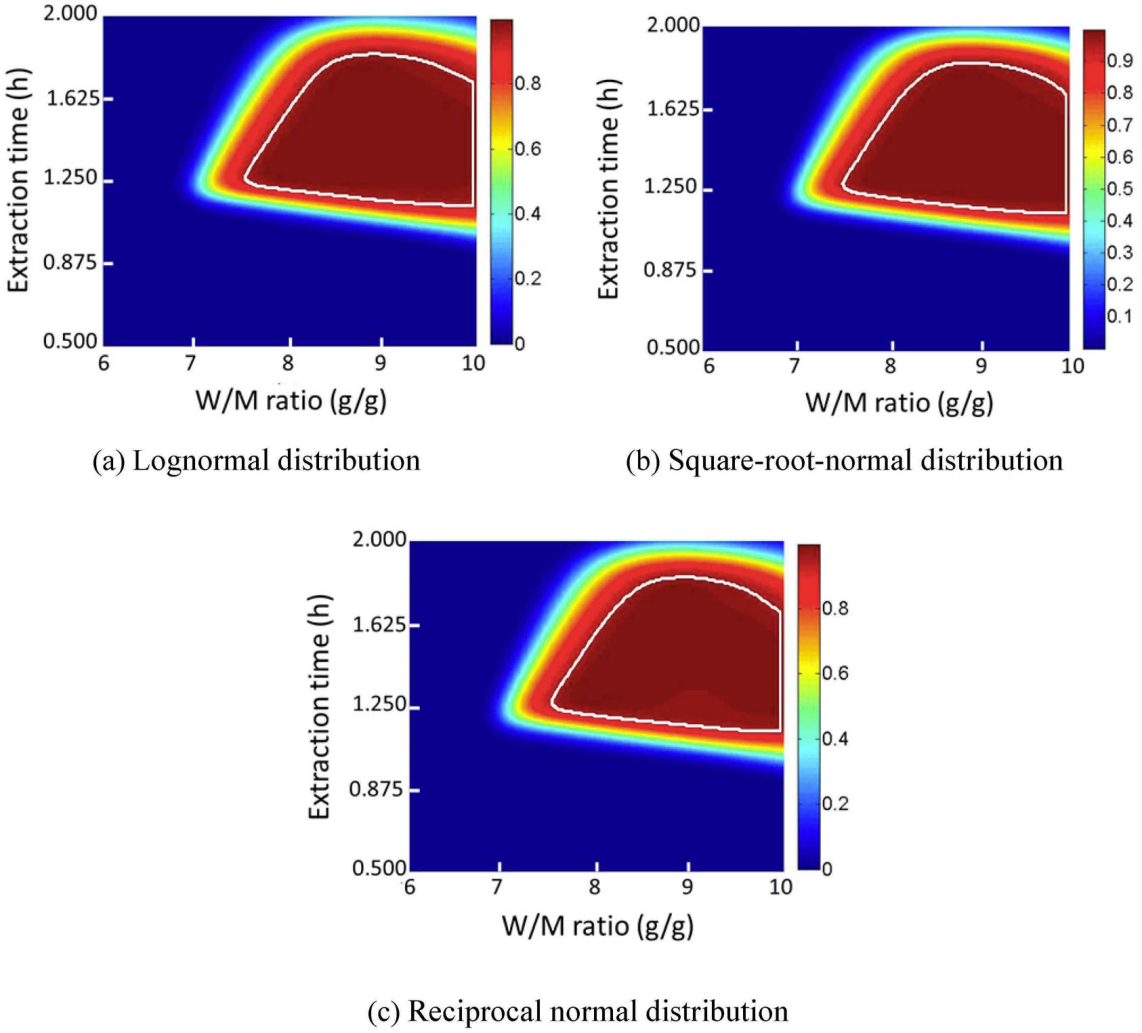
The position and shape of calculated design space with different data distribution types. (Color bar refers to the probability to attain CQA criteria; the region within the white line is the design space).

**Fig 11:**
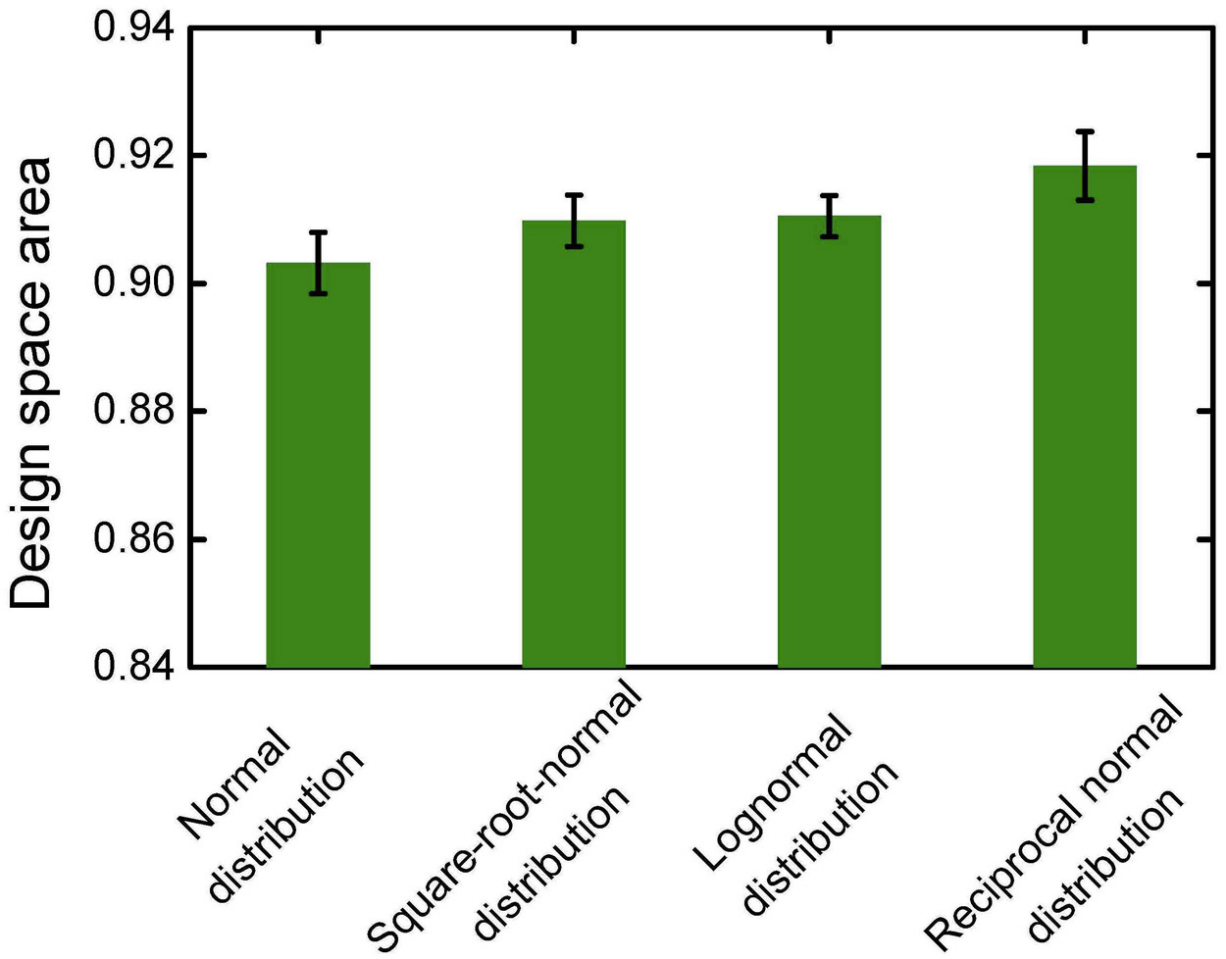
Dimensionless design space area after stepwise regression.

**Fig 12:**
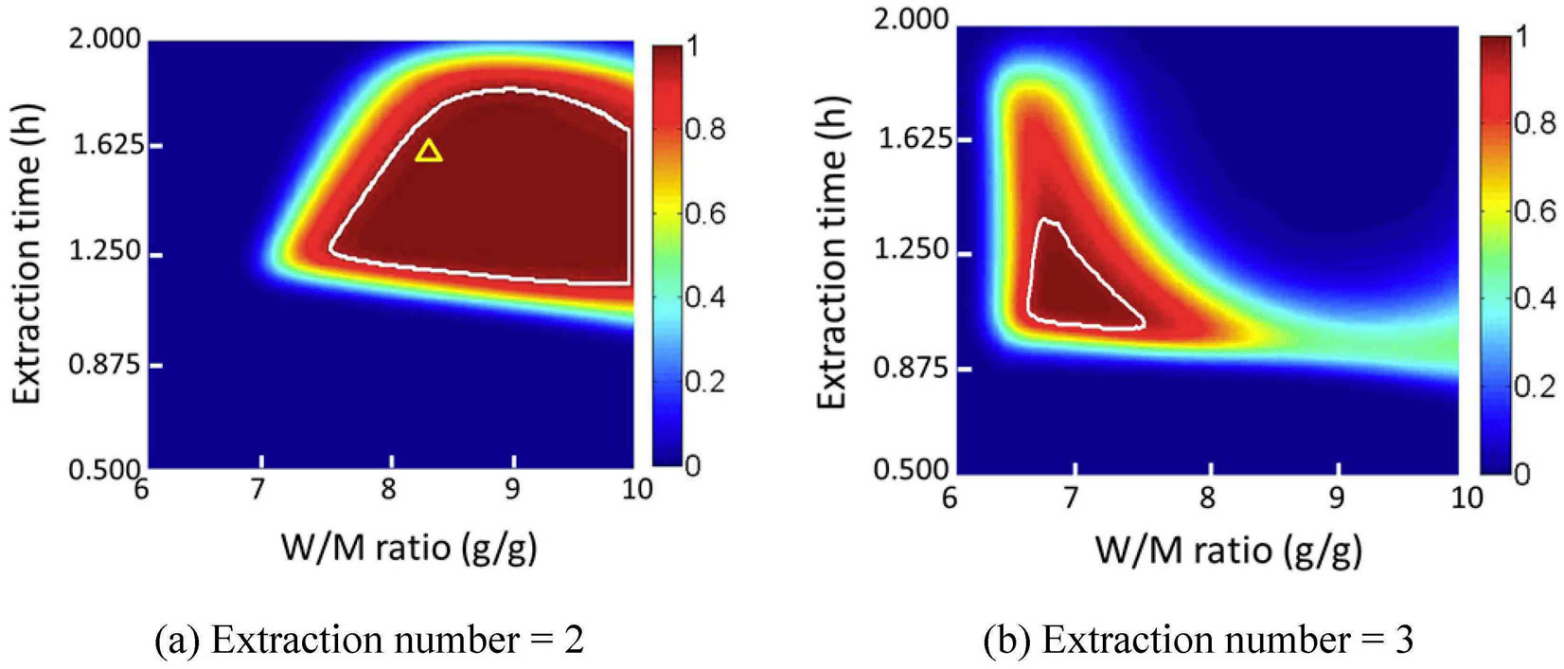
Design space and the verification experiment. (Color bar refers to the probability to attain CQA criteria; △, verification experiment; the region within the white line is the design space).

The effects of four different data distribution types on the regression results were investigated, including normal distribution, lognormal distribution, square-root-normal distribution, and reciprocal normal distribution. Average values of R^2^, R^2^_adj_, R^2^_predict_, the Akaike information criterion and the Bayesian information criterion are compared as evaluation indices. These results are plotted in Fig 9. Different distributions of data also show small effects on the average R^2^, R^2^_adj_, R^2^_predict_, Bayesian information criterion, and Akaike information criterion values. Design space location and design space area when the extraction number is 2 were also compared as indices. These results are plotted in Figs 10–12. In Figs 10 and 12a, different data distribution types show little influence on the shape and position of the design space. In Fig 11, the smallest design space was obtained when the data distribution was normal. Therefore, normal distribution is favored in design space calculation.

#### 3.4.4 Design space and verification

The optimized Monte Carlo simulation conditions were obtained as follows: the simulation number was 10000; the calculation step length was 0.01; the data were generated following a normal distribution; the significance level used in the stepwise regression was 0.35. The design space can be obtained when the extraction number is 2 or 3, as shown in Fig 12.

Considering the consumption of solvent and time, an extraction number of 2 is recommended. The recommended normal operation range is 8.2–10 g/g of W/M ratio and 1.25–1.63 h of extraction time, with a minimum probability of 0.97 of attaining CQA criteria. Design space was then verified in a larger scale extraction with 360 g of Danshen and 120 g of Honghua used with an extraction number of 2, an extraction time of 1.6 h, and a W/M ratio of 8.3 g/g. The probability of attaining CQA criteria is 0.99 under these conditions, as shown in Fig 12a. The results are listed in Table 6. Most of the experimental results agree well with the prediction results. All the results of the verification experiments are within the limits of the CQAs.

**Table 6:** Comparison of the predicted values and experimental values.

CQA	Predicted results	Experimental results	Within CQA limits
Danshensu yield (mg/g *Danshen*)	2.666	3.039 ± 0.031	Yes
Hydroxysafflor yellow A yield (mg/g *Honghua*)	6.061	5.981 ± 0.075	Yes
Rosmarinic acid (mg/g *Danshen*)	2.087	1.850 ± 0.109	Yes
Lithospermic acid (mg/g *Danshen*)	2.438	2.298 ± 0.040	Yes
Salvianolic acid B (mg/g *Danshen*)	37.40	36.78 ± 0.51	Yes
Dry matter yield (mg/g *material*)	480.3	474.8 ± 3.3	Yes

#### 3.4.5 Discussion of the present calculation method

Compared with the Bayesian method or the bootstrap method, the uncertainty of the measured data is simulated in the present method. This method is easy to understand from the perspective of classical statistics. This method is also promising in design space development for other pharmaceutical processes. However, there are several possible drawbacks. First, new data were generated from a given distribution. The assumptions to obtain the mean value and standard deviation of the given distribution facilitate calculation. However, the generated data are just a rough approximation of the actual situation and will result in some deviations in probability prediction. Second, only the RSD value of the center point is used in calculations when the other experimental conditions are not repeated. If the RSD value of the center point is not occasionally determined correctly, the calculated design space may be affected dramatically. Third, many new data sets are generated in this method. For each data set, a new equation is developed for prediction. Therefore, a large amount of computation is required. An Intel Xeon CPU (E7-4820, 2.00 GHz) was used to calculate the design space, requiring 239 min to complete the calculation at the optimized conditions. A total of 498,000 K of memory was occupied. To obtain more reliable predicted probability, multiple repetitions of the experiments for each condition are suggested to obtain more reliable mean values and standard deviations.

## Conclusions

The probability-based design space for the extraction process of the Danhong injection was developed in this work using a Monte Carlo simulation with models built using stepwise regression. The dry matter yield and the yields of Danshensu, rosmarinic acid, lithospermic acid, hydroxysafflor yellow A, and salvianolic acid B were selected as process CQAs. Extraction time, W/M ratio, and extraction number were selected as CPPs. The effects of the CPPs were investigated using a three-level experimental design. After stepwise regression, the R^2^ values of all the models are higher than 0.94. Hydroxysafflor yellow A yield increases first and then decreases as the extraction time increases. Salvianolic acid B yield decreases as extraction time increases. More active ingredients can be extracted when the extraction number increases. The increase in extraction time, extraction number, and W/M ratio all result in higher dry matter yield. The influence of the calculation step length on calculation results was small. A higher simulation number led to lower dispersion results. The smallest design space was obtained on the assumption of a normal distribution. The optimized Monte Carlo simulation conditions were obtained: normal distribution for concentration data, 10000 times for the simulation, 0.01 for the calculation step length, significance level of 0.35 for adding and removing terms in the model development. The design space for the Danhong extraction process was calculated using these conditions with a probability higher than 0.95 of attaining CQA criteria. Normal operation ranges of 8.2–10 g/g of W/M ratio, 1.25–1.63 h of extraction time, and two extractions were also calculated. Verification experiments were carried out on a larger scale. All the results are within the CQA limits, which means that the calculated design space is accurate. Determination of all the RSD values of results under different conditions is encouraged. With these RSD values used in Monte Carlo simulation, more reliable probability is expected. The use of the present method with optimized conditions to develop design space for other pharmaceutical processes appears to be promising.
